# Continuous pressure monitoring of inpatient spinal cord injured patients: implications for pressure ulcer development

**DOI:** 10.1038/s41393-022-00841-7

**Published:** 2022-08-17

**Authors:** Sarah Fryer, Silvia Caggiari, Denise Major, Dan L. Bader, Peter R. Worsley

**Affiliations:** 1 Clinical Academic Facility, School of Health Sciences, University of Southampton, Southampton, UK; 2 Salisbury NHS Foundation Trust, Salisbury District Hospital, Salisbury, Wiltshire UK

**Keywords:** Risk factors, Spinal cord diseases, Skin manifestations

## Abstract

**Study design:**

Cohort observational study.

**Objectives:**

To examine the movement profiles of individuals with spinal cord injury (SCI) during their inpatient rehabilitative phase using continuous pressure monitoring (CPM), evaluating the trends in those with skin damage.

**Setting:**

SCI specialist rehabilitation centre in the United Kingdom.

**Methods:**

Individuals with SCI (*n* = 12) were assessed using CPM in the bed and chair over a 24–72 h. Pressure data was used as a surrogate for movement using both nursing interpretation and an intelligent algorithm. Clinical features were obtained including participants age, injury level, ASIA score, co-morbidities and prescribed support surfaces. Trends between movement profiles (frequency and intervals), SCI demographics and observed skin damage were assessed using cross-tabulation and histograms.

**Results:**

The data revealed significant correlations (*p* < 0.05) between the nursing observation and algorithm for predicting movement, although the algorithm was more sensitive. Individuals with high level injuries (C1-T6) were observed to have a lower frequency of movement and larger intervals between movements when compared to low level injuries (T7-L5) during both lying and sitting periods. The individuals observed to have skin damage were predominantly those who had both a low frequency of movement and extended gaps between movements.

**Conclusions:**

Movements for pressure relief in both the bed and chair environments were dependent on the level of injury in individuals with SCI during their inpatient rehabilitation. Distinct movement patterns corresponded with those who acquired skin damage, revealing the potential clinical applications for technologies to monitor PU risk and inform personalised care.

## Introduction

Individuals with spinal cord injury (SCI) or cauda equina syndrome (CES) present with a range of motor and sensory deficits which can impair movement and put them at risk of pressure ulcers [1], These are generally dependent on the level and severity of spinal injury. Indeed, a recent study reported that 40% of SCI patients referred to rehabilitation over a 6-month period developed a pressure ulcer (PU) [2], categorised as grade 2–4 [3]. The prevalence of PUs has been observed to be associated with the level of injury [4], with corresponding values of 34% for SCI patients with tetraplegia, 47% for those with paraplegia, and 10% for hemiplegia [5]. Individuals with SCI also regularly present with impaired circulation, sensory dysfunction, respiratory problems and impaired digestion [4, 6]. In addition, these individuals often demonstrate loss of muscle tone and atrophy, causing a higher proportion of adipose tissue with poor vascular response [7]. When combined these factors created a reduction in tissue tolerance to applied skin loading [8, 9], increasing the risk of PUs. Indeed, differences in muscle and fat properties manifest as increased interface pressure, increased internal stress, and decreased perfusion in tissue under seated load [7, 10].

To reduce the risk of PUs, individuals with SCI are recommended to perform regular movements to off-load vulnerable tissues. This corresponds to movement every 2–4 h in bed and more frequently when positioned in a chair [3]. However, there is strong evidence to suggest this frequency of movement is not adhered to in the SCI population [11, 12]. Repositioning techniques known as ‘weight shifts’ are used to restore blood flow to tissues previously loaded for prolonged periods in a wheelchair [13]. However, such physically demanding strategies are often not maintained for sufficient period to ensure full tissue reperfusion [14]. In bed, repositioning between supine and lateral lying postures are recommended [3]. However, the effectiveness with which these movements are performed even with the support of experienced nurse practitioners has been questioned [15]. Pressure mapping has been well established in research and clinical practice, to measure interface pressures in patients with SCI [11]. However, it has traditionally only been used to provide a snapshot of pressure profiles [11]. Other techniques, such as accelerometers or force sensor resistors [16], have been used to track movement over prolonged periods in patients with SCI or CES [13], typically while sitting in a wheelchair. Recently, continuous pressure monitoring (CPM), up to 72 h, has been utilised to assess postures in a general population of patients in acute and community settings [17, 18].

The current authors have recently demonstrated the use of long-term pressure monitoring as a surrogate for movement, with intelligent data processing techniques in the form of an algorithm to predict both large- and small-scale movements [19]. This was limited to a few case studies of SCI patients, in which clinical observations of movement profiles were correlated to those predicted by the algorithm. Accordingly, the present study aimed to extend this monitoring in a cohort of in-patients in a specialist SCI centre. The objective was to use the monitoring technology for a prolonged period (24–72 h) at both the bed and chair interface to estimate the frequency of movement in relation to the level and nature of the spinal injury. Secondary analysis also explored trends in those who developed skin damage over the monitoring period.

## Materials and methods

This observational cohort study was designed to monitor temporal pressure parameters and movement profiles in association with pressure ulcer risk in a cohort of SCI patients. Patients were invited to participate from two wards in a specialist UK SCI centre, with informed written consent obtained prior to data collection. Both institutional and UK NHS ethics were granted for the study (IRAS-244580 and FoHS-41814). Patients were recruited into the study over a 13 month period (Jan 2019–Feb 2020), purposefully sampled from those in phase 3 of their rehabilitation. During this phase, all patients are encouraged to sit daily in their wheelchair for at least 4 h. A retrospective evaluation by the authors demonstrated that during this phase SCI individuals were at high risk of developing PUs [20]. Participants were recruited if they met the following criteria:

Inclusion criteria:

- SCI or CES.
- Regularly sat in wheelchair for more than 4 h per day (phase 3 of rehabilitation).
- Over 18 years old.
- Speak/understand English.

Exclusion criteria:

- Progressive diseases of the central nervous system (including malignant disease involving the spinal cord).
- Cerebrovascular events.
- Injuries to the brain, not including the spinal cord.
- Cases of Spina Bifida.
- Cerebral Palsy.
- Patients with major mental health disorders which may interfere with physical treatment/rehabilitation, or those sectioned under the MHA.
- Severe brain injury with a significant cognitive deficit or behavioural problems.
- Those patients with co-morbidities which may affect their ability to undertake spinal rehabilitation.

Given the heterogeneity of the participant cohort, two distinct characteristics were considered, namely, the level of injury and ASIA score. Both parameters were treated as ordinal scales according to their international definition [1].

### Data collection

To assess temporal changes in the interface pressure parameters both a bed sensing array (ForesitePT, Xsensor, Canada) and seating array (Foresite SS, Xsensor, Canada) were used. Individuals with SCI were monitored for a minimum of 24 h and a maximum of 4 days. The duration of monitoring was affected by both patient preference and their therapeutic needs. The ForesitePT consisted of a fitted mattress cover embedded with 6136 sensor cells, over a surface area of 762 × 1880 mm and spatial resolution of 15.9 mm. For seating data, the Foresite SS was fitted to the wheelchairs with 1296 sensors over a surface area of 457 × 457 mm and spatial resolution of 12.7 mm. Each system continuously recorded interface pressure values with a sampling frequency of 1 and 5 Hz for the mattress and seating sensor, respectively. Each sensor operates within the pressure range of 5–256 mmHg (0.7 −34.2 kPa), with an accuracy of ±1 mmHg. An external battery was attached to the wheelchair monitor to power the system for 12 h, to enable participants to mobilise in their chair whilst being monitored. Sensor arrays were cleaned between participant usage as per the infection prevention standards of the healthcare institution.

The lead research nurse assessed the skin of each participant three times per week for a 4 week period during and immediately after the monitoring period. Findings were documented on a standardised Pressure Ulcer Prevalence Sheet [21], including evidence of pressure ulcers or periods of bed rest for skin damage.

**Fig. 1:**
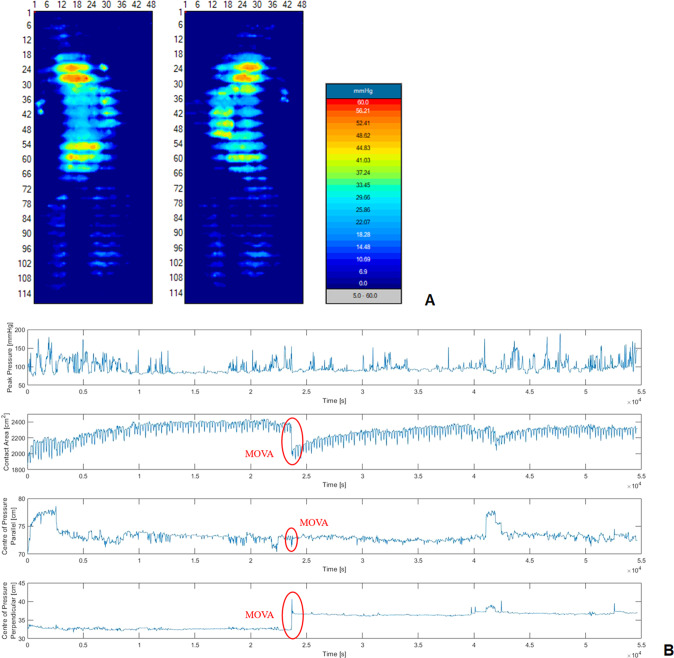
Continuous pressure monitoring data from one patient (P9) during the second night of data collection. **A** Spatial distribution of pressures from MOVA 1, corresponding to a turn from right side lying to left side lying. **B** Temporal profiles of Peak pressure, Contact Area (≥20 mmHg), Centre of Pressure. NB alternating mattress pressure signatures can be observed in the contact area plot.
(ii) An automated algorithm developed by the host lab which employs data processing and machine learning to detect movement profiles [19, 22, 23].

Two methodologies were employed to analyse the CPM data:

(i) Nursing interpretation of pressure maps, involving examining trends in key parameters, namely, centre of pressure, contact area and peak pressure, and key frame images of pressure data (Fig. 1A).

### Clinical evaluation of pressure monitoring data

The nurse interpretation was designed to identify large-scale movements, termed ‘Movements to Off-load Vulnerable Areas (MOVAs)’. Each event was defined as a movement where clear evidence of changes in the spatial distribution of pressures were achieved through changes in posture e.g. supine to lateral lying, or where a patient performs a lean in the wheelchair of sufficient magnitude and duration to off-load tissue sites, such as the ischial tuberosity. These movement were observed through retrospective analysis of the pressure data via its proprietary software (V8 Analyser, XSensor, Canada).

### Algorithm prediction

The raw data was extracted from the monitoring technologies and analysed to estimate the frequency and magnitude of MOVAs. The machine-learning approach to analyse temporal profiles of interface pressures as a surrogate for detecting changes in lying postures has been developed in the host lab and comprehensively described [19]. To review briefly, the derivative signal of combined parameters, including the centre of pressure (COP) in both planes of the sensing arrays and the contact area above a specific threshold (20 mmHg), was used to identify large-scale movements (postural changes, the latter indicated in Fig. 1B. These were selected following the analysis of the three pressure parameters using Receiver Operating Characteristic (ROC) curve analysis [22]. These parameters were subjected to a series of processing steps, which included a moving average filter with a time window of 30 samples, to remove the high frequency noise. The sum of the derivative signal was then subjected to discriminant thresholds to identify the events associated with movements. Subject-specific thresholds were established for each SCI individual, with a three-step verification performed for each movement, namely:

1. A movement was defined as a relative change in contact area (≥20 mmHg) between the current and previous posture exceeding a threshold value of 3.2%, representing the minimum change in the contact area from established data [23].
2. A static posture was defined as a posture sustained for a period exceeding 90 s, which has been reported to represent the minimum time required for soft tissues to recover from loading in SCI patients [9].

### Statistical analysis

The frequency of MOVAs per hour and largest time interval between MOVAs were estimated for each participant, using both clinical interpretation and the algorithm prediction. The trends between patient characteristics (injury level and ASIA score) and MOVA profiles were assessed using histograms and cross-tabulation. Comparison of clinical interpretation and algorithm prediction was also made using Pearson’s correlation coefficients, with significance prescribed at 5% (*p* < 0.05). Descriptive statistics were used to explore movement trends in SCI participants who had episodes of skin damage with respect to those with no skin damage.

## Results

A total of 14 SCI participants consented to take part in the study, although two withdrew prior to the monitoring period. As a result, 12 individuals completed the monitoring, with their demographics detailed in Table 1. This reveals a wide range of ages, level of injury and ASIA score, which include A, B and D. The participants presented with a series of co-morbidities, some of which may have influenced the susceptibility to skin damage e.g. diabetes mellitus (DM).

**Table 1:** Participant demographics and co-morbidities.

ID	Age (years)	SCI Level	ASIA	BMI (kg/m^2^)	Co-morbidities
P1	64	T6	A	27	Cardiac surgery
P3	75	T5	A	28	Osteoarthritis and mitral regurgitation
P4	77	T10	A	28	Thoracic AVF and pulmonary embolism
P5	66	T11	D	24	Aortic fistula and bi-iliac aneurysm
P8	70	T4	D	30	Dermatitis, asthma, hypertension
P9	53	C4	A	27	T2 DM, High cholesterol
P10	74	T11	A	21	Arthritis, high cholesterol
P11	27	C4	B	21	Asthma
P12	18	L2	B	24	ADHD, hyper reflexivity
P13	53	C5	B	28	Dental abscesses
P14	29	C8	B	25	Epilepsy
P15	30	T11	A	22	No

Each participant had been prescribed a support surface by the attending clinician based on their risk of developing a pressure ulcer, determined by their Braden score (Table 2). This table also includes information on the occurrence of bed rest due to skin damage, revealing that five of the participants (P1, P3, P5, P9 and P13) presented with skin damage during their inpatient rehabilitation. These included PUs, moisture associated skin damage (MSAD) and traumatic abrasions that did not readily heal. It is notable that only two of the participants (P1 and P9) were prescribed an alternating air mattress, with all other participants supported on castellated or non-castellated foam mattresses during their in-patient stay. In sitting, only three participants were prescribed air-based wheelchair cushions (P9, P13 and P14), with the others using foam/gel cushions.

**Table 2:** Participant support surface and skin specific features.

ID	Braden (6–24)	Wheelchair cushion	Mattress	History of bed rest
P1	14	Jay 2-Fluid/Foam	Talley Quattro-air	Yes
P3	15	Jay balance-foam/fluid	Softform Spinal-non castellated Foam	Yes
P4	17	Invacare Matrx Foam	Softform Spinal-non castellated foam	No
P5	17	Matrx libra cushion-foam	Softform premier-castellated foam	Yes
P8	16	Matrx contour-foam	Softform spinal-non castellated foam	No
P9	16	Jay 3 with Roho insert-foam/air	Talley Quattro-air	Yes
P10	15	Matrx Libra-foam/fluid	Softform premier-castellated foam	No
P11	12	Matrx libra-foam/fluid	Softform spinal-non castellated foam	No
P12	19	Mercury 300 gel	Castellated foam	No
P13	15	Starlock-air	Softform spinal-non castellated foam	Yes
P14	17	Roho Hybrid elite-air	Softform premier-castellated foam	No
P15	19	Matrx Libra-Foam	Softform Spinal-non castellated foam	no

### Nursing observations vs. algorithm predictions of movement

**Fig. 2:**
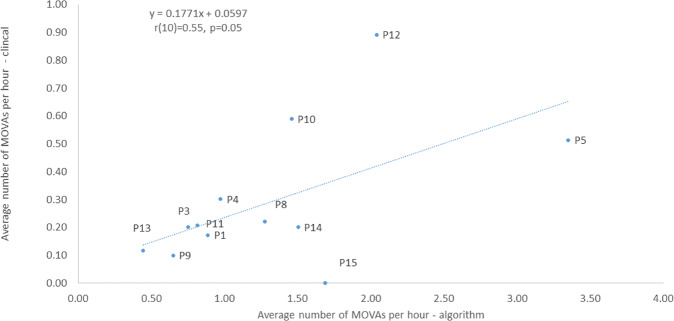
Comparison between clinical observations of MOVA frequency and the algorithm predictions from the pressure monitoring data of each participant.

Similar trends were evident when the two methods of estimating movement behaviour were compared. This is exemplified by the statistically significant correlation (*r* = 0.55; *p* < 0.05) between the two distinct estimates of the frequency of MOVAs per hour (Fig. 2). Close examination, however, revealed that the algorithm predicts higher values for the average number of MOVAs per hour than the corresponding clinical values. This may be explained by the increased sensitivity of the algorithm to detect movements, which may not have been identified by the clinician. An in-depth comparison between the algorithm and clinical observations can be found in Caggiari et al. [19]. Accordingly, the subsequent analysis has been conducted using the more sensitive algorithm data.

### Frequency and maximum intervals between MOVAs in lying

**Fig. 3:**
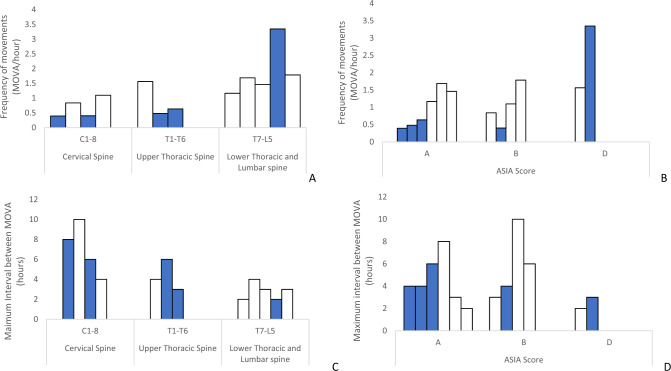
Histograms of the frequency of lying MOVA. Histograms of the frequency of lying MOVA according to **A** SCI level and **B** ASIA score. Maximum interval between lying MOVAs according to **C** SCI level and **D** ASIA score. N.B. SCI patients with skin damage are indicated with a solid bar fill.

There was a high degree of between subject variability in both the frequency of MOVA and maximal intervals between MOVA in lying (Fig. 3). The data revealed that there was a general increase in frequency of MOVAs when participants with high level lesions (C1-T6) were compared to those with lower injuries (T7-L5) (Fig. 3A). The corresponding trend was reversed with respect to the maximum intervals between lying MOVAs, with large time intervals (3–10 h) associated with participants with high level injuries and smaller time intervals between MOVAs (2–4 h) for low level injuries (Fig. 3C).

The distribution of movement parameters was similar for participants with a loss of motor sensation (ASIA B) and those with a combined motor and sensation impairment (ASIA A), with corresponding frequency ranges of 0.4–1.8 MOVAs per hour. By contrast, participants with an incomplete injury displayed a higher frequency of movement (range 1.6–3.4 MOVAs/hour). The corresponding maximum intervals between MOVAs also demonstrated distinct differences in the distribution of individuals with ASIA A/B (2–10 h) and ASIA D (2–3 h).

### Frequency and maximum Intervals between MOVAs in sitting

**Fig. 4:**
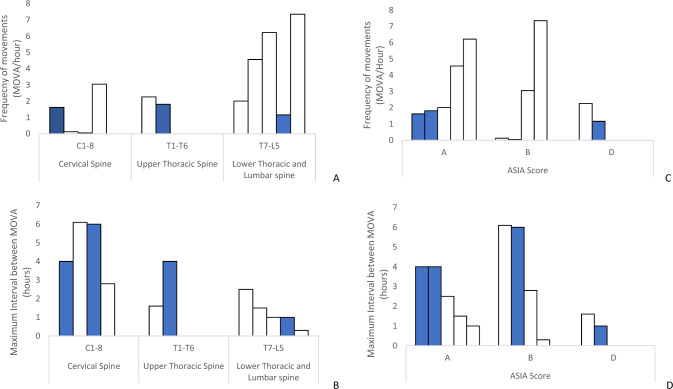
Histograms of the frequency of seating MOVA. Histograms of the frequency of seating MOVA according to **A** SCI level and **B** ASIA score. Maximum interval between lying MOVAs according to **C** SCI level and **D** ASIA score. N.B. SCI patients with skin damage are indicated with a solid bar fill.

A high degree of variability was also observed in seated MOVA profiles, with frequency ranging from 0.1 to 7.4 MOVAs per hour (Fig. 4). In a similar trend to the lying data, the results revealed that those with the highest level of injury had the lowest frequency of movement. Indeed, for each category of SCI level, the range of frequency of movement increased from 0.1–3.0 MOVAs per hour in the cervical group to 2.0–7.4 MOVAs per hour in the lower thoracic and lumbar injured individuals. There was also a notable difference in the maximum intervals between MOVA, with higher level injury participants (C1-T6) demonstrating values ranging from 3 to 6 h. By contrast, participants with a lower injury level (T7-L5) demonstrated intervals ranging from 1 to 3 h. When movement parameters were groups according to ASIA scores no differences were observed.

### Association with skin damage

A number of participants (5/12) were subjected to bed rest within the Spinal Cord Injury Treatment Centre (SCIC) due to skin damage (Table 2). It is evident that the majority of these patients presented with a high level of injury (C1-T6, *n* = 4/5) and were ASIA A or B (*n* = 4/5). Figure 3 depicts these patients also presented with a low frequency of lying movement (range 0.4–0.6 MOVAs per hour) and high maximum interval between MOVAs (range 4–8 h). By contrast, one individual with skin damage had an incomplete (ASIA D) lower thoracic injury (T11) and was observed to have the highest frequency of lying MOVA in the cohort (3.4 MOVA per hour). However, this same individual also presented with a low frequency of seated MOVA (1.16 MOVA per hour).

## Discussion

Immobility has been long recognised as an important factor in determining the risk of pressure ulcers in the SCI population. The present study aimed to characterise movement patterns in a cohort of individuals with SCI, during the rehabilitation phase of their inpatient recovery. CPM data was used as a surrogate for movement in both the bed and chair environments, analysed by nursing-led observations and the application of an intelligent algorithm. The data revealed distinct trends in movement profiles related to the level of injury and ASIA score. Movements that provide pressure relief to vulnerable tissue sites, termed MOVAs, were limited in those with high level (C1-T6) complete injuries (ASIA A and B). By contrast, regular movements were observed in those with lower-level injuries (T7-L5). It was of note, that those participants who acquired skin damage generally demonstrated very infrequent movements (Figs. 3, 4).

The present study has demonstrated how CPM coupled with an intelligent algorithm can provide critical temporal trends in posture and mobility in a cohort of individuals with SCI. Movements were estimated by both a trained clinician (SF) and an algorithm, corresponding to MOVA events which were identified through a change in the spatial distribution of pressure data [19]. These could be observed through temporal variations in key pressure parameters (Fig. 1), previously identified in lab-based assessments [22]. The MOVAs identified from the nursing observation and algorithm were compared (Fig. 2) revealing a strong correlation, albeit with a systematic increase in detection from the algorithm. Thus, the resulting data presented for each individual (Figs. 3, 4) corresponded to the algorithm with enhanced sensitivity. Indeed, through the algorithm we have a method to automatically detect and verify movement events without clinicians needing to go through the pressure monitoring data which can be time intensive and potential subjective in nature. The implications for this approach are that some of the MOVA detected by the algorithm could have been caused by smaller scale perturbations in body position, where the magnitude and duration of movement may not have been adequate to relieve previously loaded skin sites as identified in previous studies [9]. Further research is required to identify critical trends in both large- and small-scale movements which may be indicative of an individual’s risk of development pressure ulcers when combined with other intrinsic factors.

Although previous literature has investigated movement within the SCI population as a whole [11, 13], the relationship between movement and the level of injury or ASIA score has not been previously reported. Therefore, for the first time, this study addresses the movement profiles of SCI individuals while supported on both mattress and wheelchair cushion in a specialist in-patient rehabilitation setting. The monitoring corresponded to a latter phase (phase 3 or 4) of their in-patient recovery, where individuals were encouraged to spend up to 4 h sitting in a wheelchair putting them at greater risk of developing a pressure ulcer. It therefore represented a period in which greater personal responsibility for pressure care was initiated. The findings of the study revealed that those with high levels of injury i.e. cervical and upper thoracic spine present with both a reduced frequency of movement and larger intervals between movements [1]. These individuals were also observed to have the highest incidence of skin damage during the monitoring period, which corroborates previous studies on SCI groups [4]. There is international agreement that movement is one of the key interventions in PU prevention [3], limiting the time vulnerable tissues are exposed to harmful loading conditions at the skin interface. Indeed, the current study supports the use of technologies to monitor movement to enable more efficient care delivery and move to a more personalised approach for individuals with SCI [24].

SCI patients are encouraged to perform regular off-loading of tissues during prolonged periods of sitting or lying to minimise the risk of skin damage. However, a recent study found that self-reporting of pressure-relieving activities was often inaccurate [13] and, as a result, identified the need for reliable objective monitoring of pressure-relieving activities [25]. To date, these technologies can be largely categorised into two distinct approaches, namely wearable sensors involving accelerometers [25, 26] and pressure monitoring devices either at the support surface interface or placed underneath mattress/cushion systems [11, 13, 17, 27]. Although the former is recognised as a standard from which movements can be monitored, there are limitations in the compliance to wearing body-mounted sensors [28]. Further research is needed to establish the relative accuracy between actimetry and interface pressure monitoring. Indeed, for sensors placed at the support surface-individual interface, or even under the support surface itself, movement artefacts can occur [29], for example when foreign objects are placed on the bed/chair.

It was evident that the most vulnerable participants, with high injury levels (cervical spine) and ASIA scores (A or B), can be in static positions in excess of 6 h while sitting in a wheelchair (Fig. 4), which has been demonstrated in previous studies [12, 13, 30]. Direct comparisons with other studies are limited due to the method of movement monitoring, setting (most studies are in the community) and analytical approaches. However, in each case, studies identify that those with SCI are prone to prolonged periods of immobility in the bed and chair environments. During both night and day monitoring there was a wide range of movement frequencies, across the heterogenic SCI cohort. Reduced movements observed overnight may be due to a reluctance of carers to disturb the sleep of individuals, with a need to balance sleep quality and PU prevention [31]. There is no current consensus on safe levels of movement for a given individual to prevent pressure ulcers. With the tolerance to prolonged postures likely to be patient specific, depending on key factors such as age, nutrition, history of skin damage and co-morbidities [32]. There can be different risks depending on sitting or lying positions. For example, one patient (P5) who experienced skin damage, had very high frequency of movement in lying, but very low frequency of movement in sitting. Indeed, a recent observational study in which hospital patients movements were monitored with a piezoelectric motion sensor observed that pressure ulcers occurred both in low and high movers [27]. However, this study was limited to monitoring in the bed, missing the critical element of seated movements.

The data set was collected on a small cohort of in-patients with SCI, which limits its generalisability. Future studies should include evaluations of both inpatient and community settings, where it is widely established the transition to self-management can impact on the adherence to pressure ulcer prevention strategies [33]. Monitoring periods in the bed and chair inevitably varied, depending on the access to patients and their willingness to have the monitoring systems in place for prolonged periods. Therefore, direct comparison between patients is limited by the time in which the sensing array was in-situ, which could be affected by clinical routines and the health status of the individual. Direct causation between our movement observations and occurrence of skin damage is limited by the time differentiation between assessments. Indeed, in most cases skin damage did not occur during the 24–72 h CPM period, rather the 4 weeks of observations that were conducted over the proceeding period. Both the nurse-led observations and the algorithm for predicting movement from pressure parameters could be prone to some errors, with the algorithm recently demonstrated to achieve ~80% accuracy when compared to clinical observations [21]. However, the present study demonstrated a statistical correlation between the two approaches providing some confidence that key movements were identified reliably (Fig. 2). In addition, the current study focused on MOVA movements, inevitably SCI individuals also performed smaller scale ‘postural adjustments’ which would impact on skin and soft tissue health. The frequency and nature of these movements and their relative importance to PU prevention warrants further investigation. These movements can be detected using the proposed algorithm, where a two-tiered thresholding on the derivative signal being employed to differentiate between postural adjustments and larger scale movements [21]. It is of note that an air mattress was only prescribed for two participants, even though five of the cohort presented with skin damage. These small numbers precluded any specific analyses, although it is inevitable that the type of support surfaces in lying or sitting can affect the redistribution of pressures which, in turn, will influence the resulting pressure-related parameters estimated from each monitoring session.

Individuals with SCI balance the risk of developing PUs with the need for comfort and functionality, social and work-based activities. A recent systematic review and meta-analysis reported that 1 in 5 individuals with SCI will develop a PU, especially in community settings or low- and middle-income developing countries [34]. Thus, a personalised multidisciplinary approach recommended for best care [35]. Prevention should also be formed by shared decision making between the individual and their healthcare professional [33]. The present study has demonstrated that it was feasible to use CPM as a surrogate for movement in the bed and chair for individuals with SCI, identifying observations related to repositioning patterns and SCI level/ASIA score. The use of monitoring to inform personalised PU prevention strategies could create the basis of shared working between patients and healthcare workers, where objective data can be used to assess risk, identify trends that patients can also observe to form common goals for prevention strategies while in both hospitals and when transferred to the community. Indeed, studies have demonstrated how feedback from technology can promote compliance with pressure-relieving manoeuvres [30, 36]. Further research is needed to evaluate the use of monitoring and feedback strategies to support pressure ulcer prevention in individuals with SCI.

## Data Availability

The datasets generated and/or analysed during the current study are available from the corresponding author on reasonable request.
